# Cooperation between Hsp90 and mortalin/GRP75 in resistance to cell death induced by complement C5b-9

**DOI:** 10.1038/s41419-017-0240-z

**Published:** 2018-02-02

**Authors:** Perri Rozenberg, Lea Ziporen, Dana Gancz, Moran Saar-Ray, Zvi Fishelson

**Affiliations:** Department of Cell and Developmental Biology, Sackler School of Medicine, Tel Aviv University, Tel Aviv, 69978 Israel

## Abstract

Cancer cells are commonly more resistant to cell death activated by the membranolytic protein complex C5b-9. Several surface-expressed and intracellular proteins that protect cells from complement-dependent cytotoxicity (CDC) have been identified. In this study, we investigated the function of heat shock protein 90 (Hsp90), an essential and ubiquitously expressed chaperone, overexpressed in cancer cells, in C5b-9-induced cell death. As shown, inhibition of Hsp90 with geldanamycin or radicicol is enhancing sensitivity of K562 erythroleukemia cells to CDC. Similarly, Hsp90 inhibition confers in Ramos B cell lymphoma cells elevated sensitivity to treatment with rituximab and complement. C5b-9 deposition is elevated on geldanamycin-treated cells. Purified Hsp90 binds directly to C9 and inhibits zinc-induced C9 polymerization, indicating that Hsp90 may act directly on the C5b-9 complex. Mortalin, also known as stress protein 70 or GRP75, is a mitochondrial chaperone that confers resistance to CDC. The postulated cooperation between Hsp90 and mortalin in protection from CDC was tested. Geldanamycin failed to sensitize toward CDC cells with knocked down mortalin. Direct binding of Hsp90 to mortalin was shown by co-immunoprecipitation in cell extracts after triggering with complement as well as by using purified recombinant proteins. These results provide an insight into the protective mechanisms utilized by cancer cells to evade CDC. They suggest that Hsp90 protects cells from CDC by inhibiting, together with mortalin, C5b-9 assembly and/or stability at the plasma membrane.

## Introduction

The complement system takes part in the systemic immune attack on abnormal cells, such as cancer cells, and in their elimination. To achieve effective cell death, the complement system assembles the C5b-9 membrane attack complex (MAC) that inserts into cell membrane and induces cell death^1,2^. The MAC is inflicting complement-dependent cytotoxicity (CDC) via several parallel mechanisms that are still not fully characterized. Elevated calcium ion levels have been shown to play a key role in the cell death^3^. In addition, the MAC is activating a pathway of programmed necrotic cell death involving JNK and Bid^4,5^.

To resist the potential toxic effects of the C5b-9 complex, cells utilize several ubiquitously expressed membrane complement regulators, membrane cofactor protein/CD46, decay accelerating factor/CD55 and CD59^6^. Cancer cells overexpress these proteins and thus become increasingly resistant to CDC^7^. Targeting of antibodies or siRNA to these membrane regulators on cancer cells increases their sensitivity to complement attack^8,9^. Cells also actively remove the C5b-9 complexes from their surface through endocytosis and exo-vesiculation^10^. Additional protection from CDC is conferred by the mitochondrial chaperone mortalin/GRP75^11^. Mortalin is a constitutively expressed member of the heat shock protein 70 family located primarily in mitochondria and secondarily in other cellular compartments^12,13^. Elevated levels of mortalin were reported in cancer cells^14–16^. Inhibition of mortalin synthesis or activity is sensitizing cells to CDC^17^. Mortalin contributes to the shedding of C5b-9 complexes from cells in membrane vesicles and was shown to bind directly to complement C9^11,18^.

Heat shock protein 90 (Hsp90) plays essential roles in cell signaling, protein folding and maturation, and cell proliferation and survival^19–21^. Two genes encode for the cytoplasmic Hsp90, HSP90AA1 encoding for an inducible Hsp90α, and HSP90AB1 encoding for the constitutively expressed Hsp90β^22^. Two Hsp90 isoforms are located in the endoplasmic reticulum (Grp94) and the mitochondria (TRAP1)^23,24^. In cancer, Hsp90 expression is upregulated and proposed to be involved in cancer initiation and progression^25–27^. Hsp90 upregulation correlates with bad prognosis in solid tumors and leukemia^28^. Hsp90 is primarily located in the cytosol and to some extent in the nucleus. However, it is also abundant in mitochondria of cancer cells and is protective, together with TRAP1, in mitochondrial cell death^29^. Due to its numerous cellular vital functions, Hsp90 has been exploited for several years as a target in cancer therapy and small molecule Hsp90 antagonists are being examined in clinical trials^30^. Inhibition of Hsp90 was shown to confer on human cancer cells sensitivity to serum lysis^31^. Here, we have examined the protective effect of Hsp90 against CDC. The possible association between Hsp90 and mortalin in protection was investigated. Our results demonstrate that Hsp90 plays a role in cell protection from CDC and that mortalin takes part in this protection. A direct protein–protein interaction between Hsp90 and mortalin is shown, as well as an interaction between Hsp90 and complement protein C9. A role for Hsp90, in collaboration with mortalin, in the diminution of the number of C5b-9 complexes stably inserted during complement activation into the cell membrane is indicated.

## Results

### Inhibition of Hsp90 enhances cell sensitivity to CDC

**Fig. 1:**
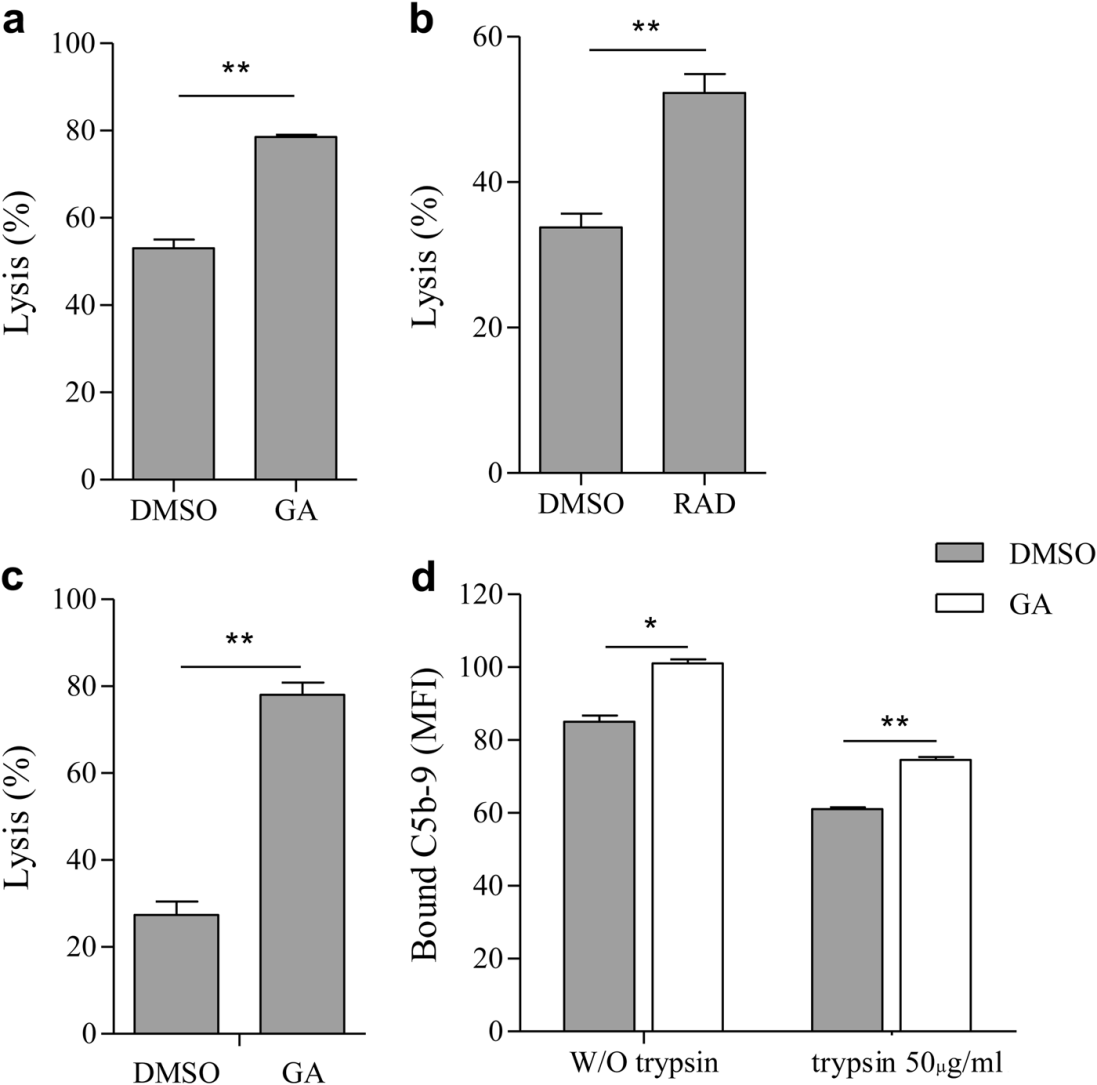
Hsp90 inhibitors sensitize cells to CDC. **a**, **b** K562 cells were incubated with geldanamycin (GA, 100 μM) (**a**) or radicicol (Rad, 100 μM) or with DMSO (0.5%) as control for 60 min at 37 °C. Then, the cells were washed and treated with antibody and NHS for 60 min at 37 °C. Percent lysis was determined by propidium iodide inclusion. **c** Ramos cells were incubated with geldanamycin (GA, 100 μM) or with DMSO (0.5%) as control for 60 min at 37 °C. Then, the cells were washed and treated with rituximab (3 μg/ml) and NHS (50%) for 60 min at 37 °C. Percent lysis was determined by propidium iodide inclusion. **d** K562 cells were treated with geldanamycin or with DMSO for 60 min, followed by antibody (30 min) and NHS (10 min, peak C5b-9 formation). The cells were then treated or not with trypsin and labeled with aE11 monoclonal antibody followed by a secondary FITC-labeled antibody. Mean fluorescence intensity (MFI) of bound C5b-9, representative of three independent experiments, is presented. *P < 0.05, **P < 0.01

The role of Hsp90 in protection of K562 cells from CDC was first examined with Hsp90 inhibitors. Cells were preincubated with either geldanamycin or radicicol or with DMSO as control for 60 min at 37 °C. The cells were then treated with rabbit anti-K562 antibody for 30 min at 4 °C and with NHS for 60 min at 37 °C. Cell lysis was determined by propidium iodide inclusion. Both geldanamycin (Fig. 1a) and radicicol (Fig. 1b) enhanced sensitivity of K562 cells to CDC. The effect of geldanamycin on sensitivity of B cell lymphocytic leukemia Ramos cells to lysis by the anti-CD20 antibody rituximab and complement was next tested. Like K562 cells, Ramos cells pretreated with geldanamycin expressed a markedly elevated sensitivity to CDC (Fig. 1c). Treatment of cells with geldanamycin or radicicol followed by antibody and heat-inactivated NHS had no effect on cell viability (data not shown).

Elevated sensitivity to CDC may result from enhanced complement activation and C5b-9 deposition. Therefore, K562 cells were treated with geldanamycin or with DMSO for 60 min, followed by antibody (30 min) and NHS (10 min, peak C5b-9 formation). The cells were then treated or not with trypsin to remove loosely bound C5b-9 and leave only membrane-inserted C5b-9 complexes, and the effect of geldanamycin on C5b-9 deposition was tested^10^. As shown in Fig. 1d, treatment with geldanamycin led to higher levels of C5b-9 deposition. This was evident both in untreated cells and in trypsin-treated cells

### Interaction between Hsp90 and complement C9 during complement attack

**Fig. 2:**
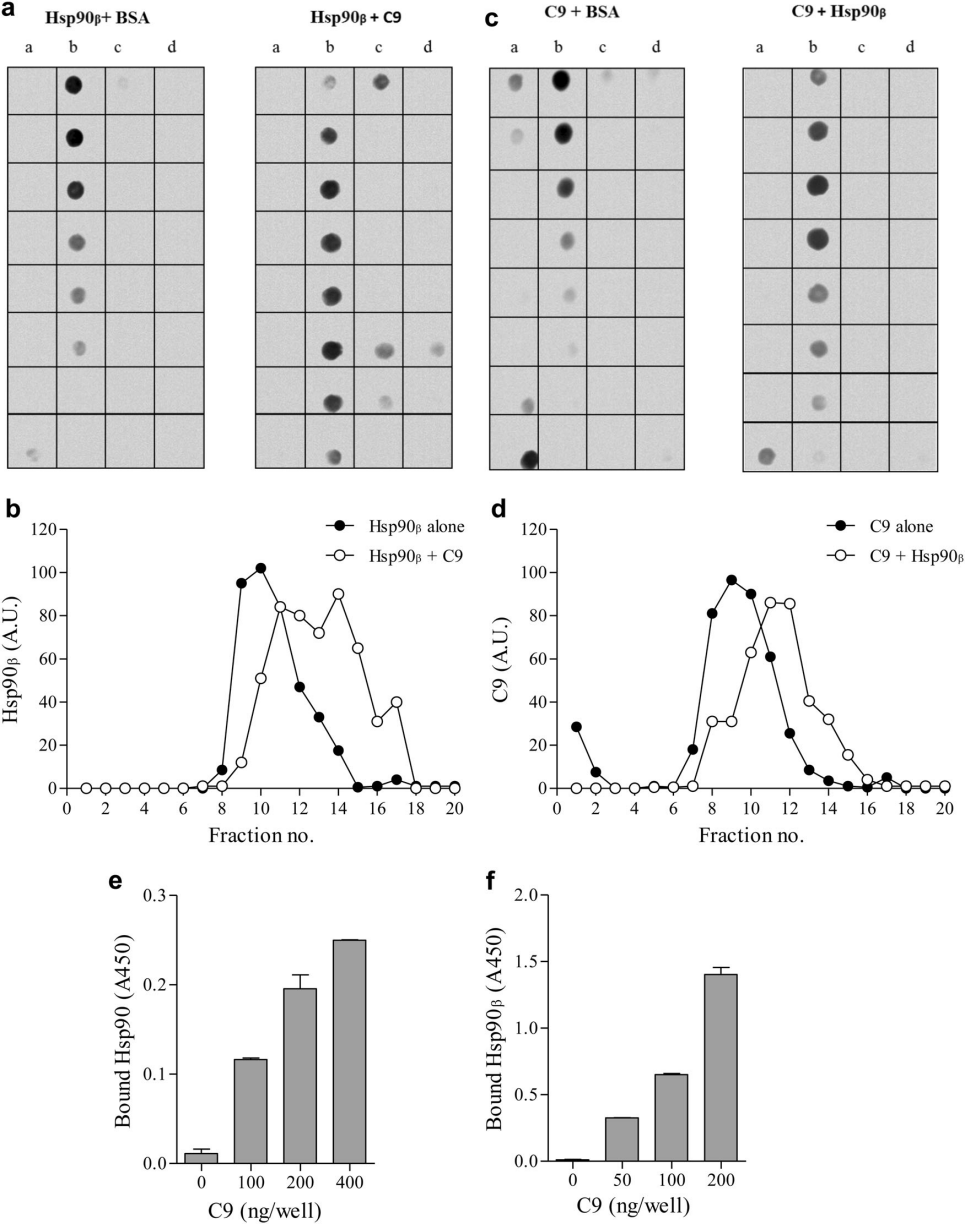
Interaction of Hsp90 with C9. **a**–**d** Hsp90β-C9 binding was tested by co-sedimentation through sucrose gradients. Recombinant Hsp90β was incubated with C9 (1 µg each) for 1 h at 37 °C. The samples were layered on top of 13 ml of 10–30% sucrose density gradients that were subjected to unltracentrifugation for 18 h at 40,000 rpm. Fractions (300 µl) were collected from the gradient top. Samples (90 µl) from each fraction were analyzed by dot blotting with anti-Hsp90 (**a**, **b**) or anti-C9 (**c**, **d**) antibody and a peroxidase-conjugated secondary antibody. Representative dot blots are shown (**a**, **c**). Density of each scanned dot was quantified in arbitrary units (A.U.). Relative distribution of Hsp90 (**b**) and C9 (**d**) in fractions 1–24 of the gradients is shown. **e**, **f** Microtiter plate wells were coated with recombinant C9 or BSA as control. Then, K562 cells lysates (**e**) or recombinant Hsp90β (**f**) were added to the wells for 60 min at 37 °C. Binding of cytosolic (**e**) and recombinant (**f**) Hsp90 was quantified (optical density, A450) with anti-Hsp90 antibodies, followed by peroxidase-conjugated secondary antibodies. Increasing quantities of C9 yielded higher Hsp90 binding (*P* < 0.001, one-way ANOVA)

Earlier findings of direct binding between mortalin and C9^11,18^ raised the possibility of a direct interaction between Hsp90 and C9. Binding of purified Hsp90β (the constitutively expressed form of Hsp90) to C9 was first examined by using density gradient centrifugation, as described under “Methods” section. Hsp90β was mixed with C9 or with BSA for 1 h at 37 °C. Each sample was layered on top of a sucrose density gradient (1–20% sucrose) and subjected to ultra-centrifugation. Fractions were collected from the top of the gradient and samples from each fraction were analyzed by dot dotting with antibodies directed to Hsp90β (Fig. 2a, b) or to C9 (Fig. 2c, d). As shown, Hsp90β and C9 mixed together reached denser fractions of sucrose, than when mixed with BSA, indicating that the two proteins interacted with each other and formed heavier complexes.

Direct binding of Hsp90 and C9 was also tested in an ELISA. First, microtiter plate wells were coated with C9 or BSA. Then, K562 cell extracts were added to all wells for 1 h at 37 °C. Binding of cellular Hsp90 was quantified with monoclonal mouse anti-Hsp90 antibody and a peroxidase-conjugated goat anti-mouse IgG antibody. Non-specific binding of Hsp90 to BSA was quantified and subtracted from the binding to C9. A significant dose-dependent specific binding of cellular native Hsp90 to C9 was observed (Fig. 2e). In addition, microtiter plate wells were coated with C9 or BSA and binding of recombinant Hsp90β was quantified as above. Non-specific binding of Hsp90β to BSA was subtracted from the binding to C9. Again, a significant dose-dependent binding of C9 to Hsp90β was observed (Fig. 2f).

**Fig. 3:**
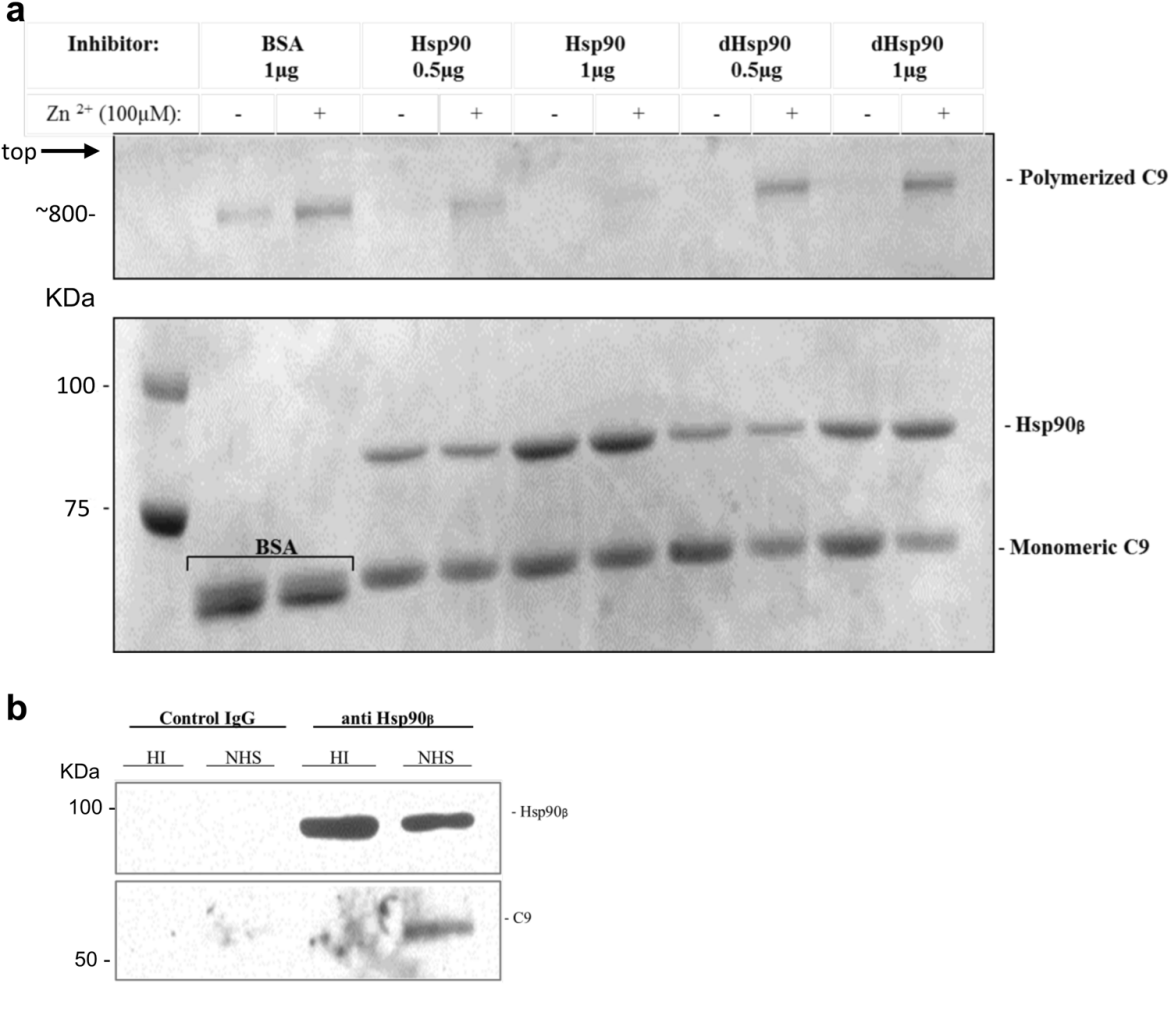
Further indications for Hsp90-complement C9 interaction. **a** Hsp90 inhibits C9 polymerization. C9 (1 µg) was mixed with recombinant Hsp90β or heat (60 °C) denatured Hsp90β (dHSP90), or BSA as control (1 µg) and then incubated with or without ZnCl2 for 2 h at 37 °C. The samples were subjected to SDS-PAGE on a 3–10% acrylamide gradient gel and stained with Coomassie blue. The bands of poly C9, monomeric C9, and Hsp90β are indicated. **b** Cell lysates after complement activation contain Hsp90–C9 complexes. K562 cells were incubated with a sublytic dose of anti-K562 antibody and with normal human serum (NHS) or heat-inactivated (HI) serum for 10 min at 37 °C. Cell lysates were prepared and then immunoprecipitated with mouse anti-Hsp90 antibody (or control mouse IgG) coupled to agarose beads. Proteins attached to the beads were examined by SDS-PAGE and western blotting. Detection was with anti-Hsp90 (upper) and anti-C9 (lower) antibodies

The possibility that Hsp90, acting as a chaperone, is directly affecting C5b-9 complex formation was raised. The last step in the process of C5b-9 complex formation is C9 polymerization. This step can be recaptured in the tube in the absence of the C5b-8 complex by incubating purified C9 with zinc ions for 2 h at 37 °C^32^. The effect of recombinant Hsp90β on C9 polymerization was tested. As control, we used heat-denatured (15 min at 60 °C) Hsp90β (dHsp90). Indeed, the results clearly showed that Hsp90β, but not denatured Hsp90β, inhibited zinc-induced C9 polymerization (Fig. 3a). BSA was added as another control for Hsp90β and had no effect on C9 polymerization.

Finally, the postulated interaction of Hsp90 with C9 in cells exposed to a complement attack was examined by co-immunoprecipitation. Cell extracts prepared from K562 cells treated with a sublytic dose of antibody and NHS or HIS (10 min at 37 °C) were precipitated with anti-Hsp90β antibodies or normal mouse IgG. The amount of immunoprecipitated C9 was quantified by western blotting. Hsp90–C9 binding was found in extracts of complement-treated cells after precipitation with anti-Hsp90β antibodies, but not in HIS-treated cells nor after precipitation with normal mouse IgG (Fig. 3b).

### Cooperation between Hsp90 and mortalin in cell protection from CDC

**Fig. 4:**
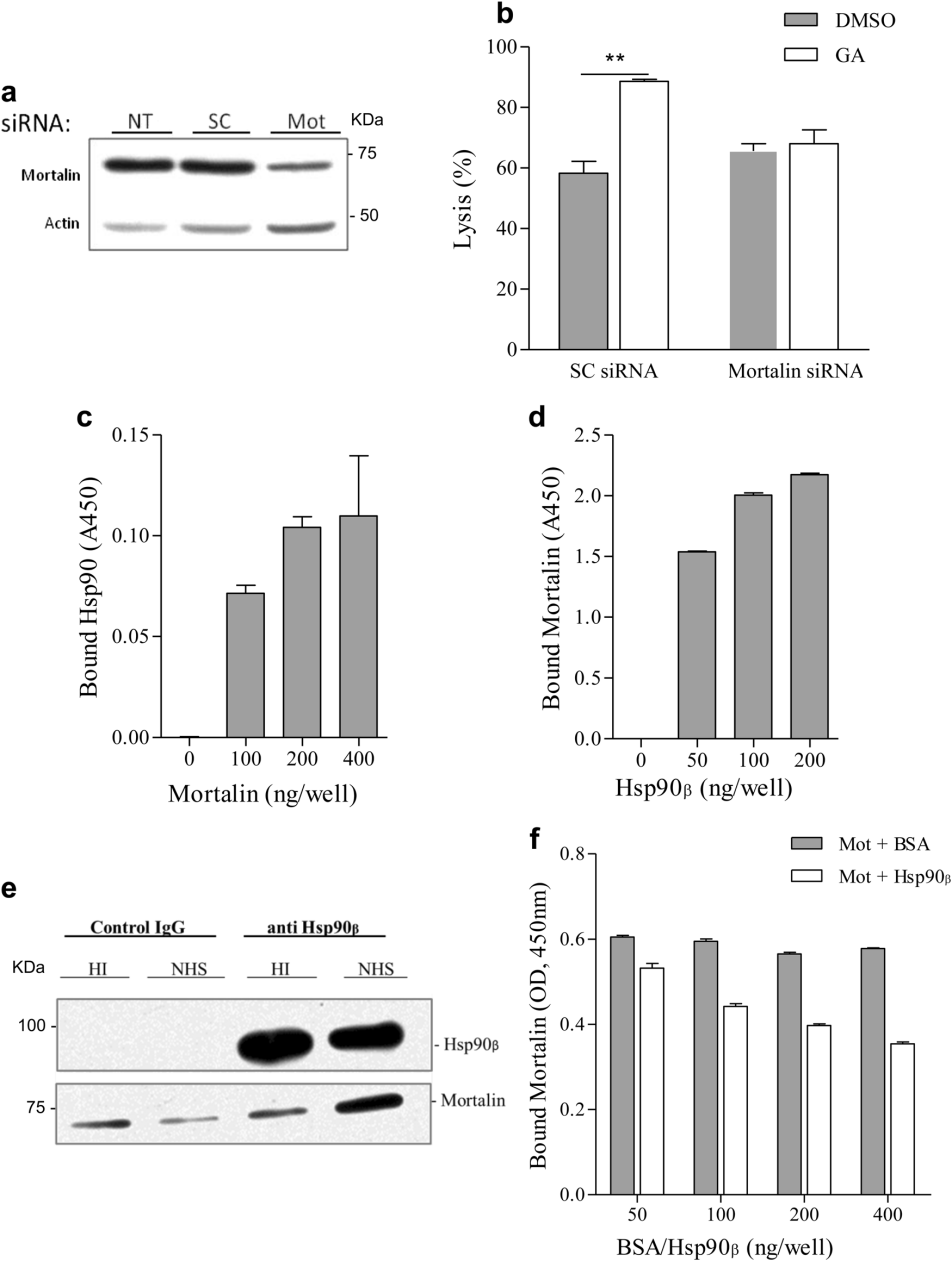
Collaboration between mortalin and Hsp90 in complement resistance. **a**, **b** K562 cells were transfected with mortalin siRNA (Mot) or a non-specific siRNA (SC). After 48 h, the level of mortalin in the cells was examined by SDS-PAGE and western blotting with anti-mortalin antibody and with anti-actin antibodies (**a**). Transfected cells were treated with GA (100 μM) or DMSO and then subjected to treatment with antibody and NHS. Percent lysis was determined by propidium iodide inclusion (**b**). NT non-treated. **c**, **d** Microtiter plate wells were coated with recombinant mortalin (C) or Hsp90β (D) or BSA as control (0). Then, K562 cells lysates (**c**) or recombinant mortalin (**d**) were added to the wells for 60 min at 37 °C. Binding of Hsp90 (**c**) was quantified (optical density, A450) with anti-Hsp90 antibodies and binding of His-tagged mortalin (**d**) was detected with anti-His antibodies, both followed by peroxidase-conjugated secondary antibodies. A dose-dependent binding of Hsp90 and mortalin was observed (*P* < 0.05 (**c**), *P* < 0.001 (**d**), one-way ANOVA). **e** K562 cells were incubated with a sublytic dose of anti-K562 antibody and with normal human serum (NHS) or heat-inactivated (HI) serum for 10 min at 37 °C. Cell lysates were prepared and then immunoprecipitated with mouse anti-Hsp90 antibody (or control mouse IgG) coupled to agarose beads. Proteins attached to the beads were examined by SDS-PAGE and western blotting. Detection was with anti-Hsp90 (upper) or anti-mortalin (lower) antibodies. **f** Microtiter plate wells were coated with C9 (100 ng/well) overnight. Mortalin was mixed with Hsp90β or BSA and added to the wells. Binding of His-tagged mortalin to C9 was quantified with anti-His (mortalin) antibodies and peroxidase-conjugated secondary antibodies. Mortalin–C9 binding was significantly inhibited by Hsp90β but not by BSA (*P* < 0.001, two-way ANOVA)

Mortalin also plays a role in cell protection from CDC^17^. We examined whether it acts together with Hsp90 in the same protective pathway. If Hsp90 and mortalin act together, geldanamycin should have no effect on CDC after knocking down of mortalin. K562 cells were transfected with mortalin siRNA or with a scrambled siRNA. The expression level of mortalin in cells transfected with mortalin siRNA was indeed reduced (Fig. 4a). As can be seen in Fig. 4b, geldanamycin increased sensitivity to CDC of cells transfected with scrambled siRNA. In contrast, sensitivity of cells with knocked down mortalin to CDC was not affected by treatment with geldanamycin.

Direct binding of Hsp90 and mortalin was tested in an ELISA. After coating microtiter plate wells with recombinant mortalin or BSA, K562 cell extract was added to the wells for 1 h at 37 °C. After washes, Hsp90 in wells was quantified with mouse anti-Hsp90 antibodies and peroxidase-conjugated goat anti-mouse IgG antibody. Non-specific binding of Hsp90 to BSA was also quantified and subtracted from the binding to mortalin. A significant dose-dependent binding of cellular native Hsp90 to mortalin was observed (Fig. 4c). In addition, microtiter plate wells were coated with recombinant Hsp90β or BSA and binding of recombinant His-tagged mortalin was quantified. Non-specific binding of mortalin to BSA was subtracted from the specific binding to Hsp90β. Again, a significant dose-dependent binding of mortalin to Hsp90β was observed (Fig. 4d).

We next examined whether C5b-9-induced stress activates an association between mortalin and Hsp90. In support, mortalin–Hsp90 binding was demonstrated in K562 cells by co-immunoprecipitation after complement activation for 10 min (Fig. 4e). A small amount of mortalin was retained on agarose beads coated with normal mouse IgG. However, the quantity of mortalin immunoprecipitated with anti-Hsp90 antibodies markedly increased in cells subjected to a sublytic dose of antibody and complement (NHS).

Competition between mortalin and Hsp90 on binding to C9 was tested in a competitive ELISA. Microtiter plate wells were coated with C9. Then, recombinant mortalin pre-mixed with increasing amount of Hsp90β or BSA was added to each well for 1 h at 37 °C. Binding of mortalin was quantified with monoclonal mouse anti-mortalin antibody and a secondary peroxidase-conjugated goat anti-mouse IgG antibody. The results clearly showed that Hsp90β competed with mortalin, in a dose-dependent manner, on binding to C9 (Fig. 4f). BSA had no effect on mortalin–C9 interaction.

## Discussion

As shown here, Hsp90 protects K562 cells from necrotic cell death activated by antibody and complement. Complement-dependent cytotoxicity of K562 cells is markedly enhanced by inhibition of Hsp90 with geldanamycin or radicicol. Geldanamycin and radicicol that act as ATP mimetics inhibit Hsp90 by binding to its ATPase site^33,34^. Hsp90 is a major cytosolic chaperone, yet in cancer cells it accumulates also in mitochondria^29^. This raises the possibility that both cytosolic and mitochondrial Hsp90 are involved and perhaps cooperate in protection of cells from the damages inflicted by the complement C5b-9 complexes. Indeed, mitochondrial damage is one of the earlier intracellular damages observed in cells undergoing CDC^35^. Mitochondrial Hsp90 was shown to confer upon cancer cells cell death resistance by suppressing the mitochondria-initiated calcium-mediated interorganelle stress response^36^. It is reasonable to assume that Hsp90 similarly protects mitochondria from CDC in cells injured by membrane-bound lytic C5b-9 complexes.

A major mitochondrial chaperone that regulates cell stress induced by complement is mortalin^11,17,18^. Inhibition of mortalin with MKT-077 or by mRNA knockdown with specific siRNA amplifies cell sensitivity to CDC. Dual targeting of mortalin and Hsp90 was shown to increase the efficacy of cancer cell death by enhancing p53-mediated apoptosis in hepatocellular carcinoma cells^37^. Binding of mortalin to the Hsp90 paralog in the endoplasmic reticulum Grp94 was reported^38^. Our results demonstrate cooperation between Hsp90 and mortalin in cell protection from complement attack. Upon knocking down of mortalin in K562 cells with siRNA, the cells become insensitive to CDC promoting activity of geldanamycin (Fig. 4b). Thus, Hsp90 inhibition is effective as long as mortalin is present. In its absence, the cells have alternative means of protection from CDC^39^. Interestingly, even though partial knockdown of mortalin by siRNA did not significantly increase percent cell death (Fig. 4b), it completely abrogated the Hsp90 protective activity. This suggests that Hsp90 anti-CDC protective activity is lost in the absence of mortalin, whereas, in contrast, mortalin has Hsp90-dependent and Hsp90-independent anti-CDC protective activities. A more complete knockdown or specific inhibition of mortalin markedly enhances K562 cell sensitivity to CDC^17^.

For functional cooperation, do mortalin and Hsp90 bind to each other? As shown here by co-immunoprecipitation analysis (Fig. 4e), control cells show almost no mortalin–Hsp90 binding. However, a clear mortalin–Hsp90 interaction occurs within 10 min after a complement stress. Direct binding has also been confirmed with purified mortalin and Hsp90β (Fig. 4c, d). It still remains to be determined where in the cell do mortalin and Hsp90 associate and which other proteins and signals are involved in this interaction. Hsp90 chaperones play vital and pivotal roles in several cellular compartments and activities^19,20^. They interact with numerous co-chaperones and client proteins that include other heat shock proteins, kinases, phosphatases, transcription factors, and other proteins^40^. The list of annotated interactions is extensive as presented in Picard’s table of Hsp90 interactors (http://www.picard.ch/downloads/Hsp90interactors.pdf) and Altieri’s mitochondrial Hsp90 proteome analysis^41^. Mortalin can be added now to the list as a stress-associated Hsp90 binder. Since mortalin binds to Hsp60 too^42^, it is likely that for regulation of certain stress conditions, mortalin joins the mitochondrial multi-chaperone complex comprised of HSP60 and HSP90^43^.

For protection from CDC, cells continuously remove the C5b-9 complexes from their surface, a process that depends on mortalin^11,17,18^. Mortalin inhibition is leading to elevated C5b-9 deposition. Similarly, inhibition of Hsp90 with geldanamycin is also amplifying C5b-9 deposition on K562 cells (Fig. 1). Direct binding of Hsp90β to C9 was demonstrated in cell lysates and with purified proteins by using several techniques (Figs. 2 and 3). Like mortalin^17^, Hsp90β inhibits polymerization of purified C9 induced by zinc ions (Fig. 3a). Interestingly, Hsp90β competes with mortalin on binding to C9 (Fig. 4f), suggesting that they both bind to the same site in C9. Concomitant binding of both Hsp90 and mortalin to C5b-9 may be envisaged as it contains an oligomer composed of several C9 molecules. In complement-triggered cells, Hsp90β and mortalin bind to each other and both are required for efficient protection from CDC. In support, protection was not achieved by geldanamycin in mortalin knockout cells. Hence, it is conceivable that C5b-9-induced formation of Hsp90–mortalin hetero-complexes is a prerequisite for efficient protection against C5b-9. Whether or not the major protective activity of the Hsp90–mortalin complexes is targeted at the C5b-9 complexes in the plasma membrane, still remains to be determined.

In conclusion, programmed necrotic signals activated by complement C5b-9 are apparently downregulated by several members of the heat shock protein family, including Hsc70^44^, mortalin^18^, and as shown here, Hsp90. These chaperones are overexpressed in cancer, and thus act as major obstacles to attempts to achieve cancer cure by antibody/complement-based immunotherapy. Currently, major attempts are being made to use chaperones inhibitors as adjuvant therapy to chemotherapy. Blocking of these chaperones in cancer will likely also amplify the efficacy of anti-cancer therapeutic antibodies through enhanced complement-dependent cytotoxicity. Hence, it is proposed that cancer patients receiving antibody therapy may benefit from co-administration of an Hsp90 inhibitor. Furthermore, the findings open new venues for interference with CDC in clinical cases in which complement C5b-9 is pathogenic (e.g., antibody-based autoimmune and inflammatory diseases).

## Material and methods

### Cells, sera, and reagents

K562 human erythroleukemia cells and Ramos B cell lymphocytic leukemia cells were cultured in RPMI-1640 supplemented with 10% fetal calf serum, 2% sodium pyruvate, 1% glutamine, and 0.2% antibiotic–antimycotic mixure at 37 °C and 5% CO_2_. The cells were split every 24–48 h so that all experiments were performed with cells in their logarithmic growth phase. Normal human serum (NHS) was used as a source for complement. Venous blood drawing from normal volunteers received approval of the Ethical Committee of Tel Aviv University. Serum was prepared from the blood and stored immediately in aliquots at −70 °C until used. Heat-inactivated serum (HIS) (45 min at 56 °C) was also prepared and used as a negative control. C8-deficient human serum (C8D) was prepared from a C8β-deficient patient as described^45^. Purified human C9 and recombinant human Hsp90β were purchased from Complement Technology Inc. (Tyler, TX) and StressMarq (Victoria, Canada), respectively. Recombinant human mortalin with a 6xhis-tag was prepared as described before^17^. Geldanamycin (from *S. hygroscopicus*), radicicol was purchased from Sigma-Aldrich (St Louis, MO).

### Antibodies

Polyclonal antiserum directed to K562 cells was prepared in rabbits and polyclonal anti-human C9 antiserum was prepared in goats. This received approval of the Tel Aviv University Animal Committee and the Israel Ministry of Health. Rituximab (MabThera) antibody was purchased from Roche Pharmaceuticals, Israel. Mouse monoclonal antibody anti-GRP75 (mortalin) and mouse monoclonal antibody anti-Hsp90β were from StressMarq. Mouse monoclonal antibody anti-Hsp90 was from StressGen (Farmingdale, NY). Mouse monoclonal antibody anti-6xHis was from Clontech (Mountain View, CA). Mouse monoclonal antibody anti-human C5b-9 (clone aE11) was from Dako (Glostrup, Denmark). Mouse monoclonal antibody anti-actin was from Millipore (Billerica, MA). Mouse IgG was purchased from Sigma (Rehovot, Israel). Peroxidase-conjugated goat antibody anti-mouse IgG, peroxidase-conjugated rabbit antibody anti-goat IgG and FITC-conjugated goat antibody anti-mouse IgG were from Jackson ImmunoResearch Laboratories (Baltimore, MD).

### Cell death measurement

K562 or Ramos cells (1 × 10^6^) were treated with rabbit anti-K562 antibody or rituximab, respectively, diluted in phosphate-buffered saline containing 1 mM CaCl_2_ and 1 mM MgCl_2_ (PBS) for 30 min at 4 °C. Next, NHS was added (final 50%) for 60 min at 37 °C. The cells were washed by centrifugation (250 × *g*, 8 min, 4 °C) and suspended in PBS containing 1 µg/ml propidium iodide (PI). The cells were analyzed by flow cytometry in a FACSort (Becton Dickinson, San Jose, CA). The percentage of dead cells (PI labeled cells) was determined (WINMDI software) and the percent of dead cells after antibody and HIS treatment was subtracted from that of antibody and NHS-treated cells. Cell lysis was also determined microscopically by trypan blue exclusion (0.02%). Mouse fibroblasts were subjected to cytotoxicity assays after trypsinization. Unlike human cells, mouse cells activate complement in the absence of exogenous antibodies. Therefore, fibroblasts cell death assays were performed with diluted NHS without added antibodies.

### Measurement of C5b-9 deposition and C9 polymerization

K562 cells were treated with rabbit anti-K562 antibodies for 30 min at 4 °C and then with NHS for 10 min at 37 °C. To avoid adverse effects of cell death, a sublytic dilution of the antibodies (yielding 10–20% cell death) was used. This was followed by treatment with trypsin (trypsin-TPCK, Sigma, 50 µg/ml) (to remove loosely attached C5b-9 complexes) or with PBS for 20 min at room temperature. For C5b-9 labeling, the cells were treated with aE11 monoclonal antibody^46^, followed by a secondary FITC-conjugated antibody. Cells were analyzed by flow cytometry (Becton Dickinson, San Jose, CA). Mean fluorescence intensity of cells labeled with aE11 was determined with the WinMDI software.

Purified human C9 (1 µg) was incubated in Tris-HCl buffer (20 mM, pH 7.2) containing with 100 µM ZnCl_2_ for 2 h at 37 °C^32^. The samples were reduced with dithiothreitol (150 μM) and subjected to SDS-polyacrylamide gel electrophoresis on a 3–10% acrylamide gradient gel. The gel was stained with Coomassie blue.

### Cell extracts

K562 cells treated with antibody and complement under sublytic conditions, as described above, were spun down by centrifugation after 10 min incubation in the NHS. For preparation of cell extracts, the cells were washed with HBSS (Sigma) and mixed with lysis buffer (0.77% Triton X-100 in 100 mM Tris, pH 7.5, 40 µl/ml protease inhibitor cocktail (“Complete”, Roche, Mannheim, Germany), 10 mM EDTA) and incubated for 30 min at 4 °C (1 × 10^6^ cells/ml lysis buffer). Next, the lysed cell extract was cleared by centrifugation (18,000 × *g*, 15 min, 4 °C) and the supernatant was collected and kept frozen at −80 °C.

### RNA interference

K562 cells were transiently transfected with siRNA by electroporation. Cells were suspended in electroporation buffer (20 mM PIPES, pH 7.0, 128 mM potassium glutamate, 10 µM calcium acetate, 2 mM magnesium acetate in RPMI growth medium). The siRNA was added first into sterile electroporation cuvettes (Cell projects, Kent, UK) and then the cells were added and kept on ice for 10 min. Following electroporation in a BTX ECM 830 Electroporator (Harvard Apparatus, Holliston, MA) (1500 µF, 250 mV), the cells were grown for 2 days in their culture medium before examination. Human mortalin siRNA (AUUGUAUUCUCCGAGUCAGUU) and a non-specific siRNA (ACUCUAUCUGCACGCUGACUU) (Dharmacon, Lafayette, CO) were used.

### Western blotting

Proteins were diluted in sample buffer (187 mM Tris-HCl, pH 6.8, 30% glycerol, 9% SDS, 1% Bromophenol blue), heated at 95 °C for 5 min, and then subjected to SDS-polyacrylamide gel electrophoresis (SDS-PAGE) over 10% or 3–10% acrylamide. Next, the proteins were transferred from the gel to a nitrocellulose membrane and the membrane was blocked with 5% skim milk in TBS for 1 h in room temperature. Then, the membranes were incubated with mouse anti-mortalin or anti-Hsp90β antibody in TBS containing 1% skim milk overnight at 4 °C. The membrane was washed with TBS containing 0.5% Tween-20 and incubated with a secondary peroxidase-conjugated goat anti-mouse IgG antibody for 1 h at room temperature. Finally, the membrane was reacted with Supersignal West Pico Chemiluminescent Substrate (Pierce, Rockfold, IL) for 5 min and exposed to an X-ray film. The optical density of the bands was quantified after scanning by analysis with the ImageJ software (NIH, Bethesda, MD).

### Analysis of protein interactions by ELISA

Wells of 96-well plate (Nunc, Thermo Fisher Scientific, Rochester, NY) were coated with purified C9 or mortalin or bovine serum albumin (BSA, Sigma) in TBS buffer (10 mM Tris-HCl, pH 7.5, 150 mM NaCl) overnight at 4 °C. The wells were washed with TBS containing 0.5% Tween-20 and blocked for 1 h at 37 °C with TBS containing 1.5% BSA. Next, either mortalin or C9 or cell lysate was added for 1 h at 37 °C. After washing, the first detecting antibody was added for 1 h at 37 °C. The secondary peroxidase-labeled antibody was then added for 1 h at room temperature. Binding was quantified with TMB One Component Microwell substrate and TMB Stop solution (Southern Biotech, Birmingham, AL) and analyzed in an ELISA plate reader at 450 nm.

### Analysis of protein interactions by density sedimentation

Proteins in TBS buffer (10 mM Tris-HCl pH 7, 150 mM NaCl) were mixed and incubated for 1 h at 37 °C. Then, the proteins were layered on top of 13 ml of 1–20% sucrose gradient in TBS in an ultra-centrifugation nitrocellulose tube and subjected to centrifugation (132,000 × *g*, 18 h, 4 °C) in a Beckman Ultracentrifuge (Fullerton, CA). Fractions (0.3 ml) were collected from the top of the gradient using Auto Densi-Flow fractionator (Labconco, Kansas city, MO) and a Pharmacia Biotech RediFrac fraction collector. Samples (80 μl) from each fraction were blotted onto a nitrocellulose membrane in a Bio-Dot Microfiltration Apparatus (Bio-Rad, Hercules, CA) and proteins were detected with antibodies directed either to Hsp90β or C9, followed by peroxidase-conjugated secondary antibodies and development with Supersignal West Pico Chemiluminescent Substrate. The optical density of the dots was quantified as described for western blotting.

### Analysis of protein interactions by co-immunoprecipitation

K562 cell lysates were first treated overnight with protein A/G agarose beads (Santa Cruz Biotechnology, Santa Cruz, CA) in TBS at 4 °C. All procedures described were performed on a rotating platform. Then, the agarose beads were removed from the cell lysates by centrifugation. In parallel, fresh protein A/G agarose was incubated overnight with either monoclonal mouse anti-Hsp90β or with mouse IgG. Then, the antibody-bearing beads were blocked with 3% BSA in TBS for 1 h at room temperature. Next, the pre-cleared lysates were mixed with the antibody-coated beads and incubated for 2 h at room temperature. The beads were washed four times with TBS, suspended in SDS-PAGE sample buffer, warmed to 95 °C for 5 min, and bound proteins were removed from the beads for analysis by SDS-PAGE and western blotting.

### Statistical analysis

The experiments were conducted in duplicates, each repeated at least three times. The results are presented as means ± SD. Statistical significance was determined by using the two-tailed and unpaired Student's *t* test. Multiple group comparison was calculated by one-way ANOVA. Two-way ANOVA was performed to compare two groups, a tested reagent and the interaction between them. GraphPad Prism 5 was used. Statistical significance was assumed when the *P* value was smaller than 0.05.
